# Geographical location and cultivar‐linked changes on chemical properties of olive oils from Algeria

**DOI:** 10.1002/fsn3.2810

**Published:** 2022-04-21

**Authors:** Said Touati, Smail Acila, Dalenda Boujnah, Hechmi Chehab, Mohamed Ayadi, Mohamed Debouba

**Affiliations:** ^1^ Higher Institute of Applied Biology of Medenine University of Gabes Tunisia; ^2^ Department of biology Faculty of Natural and Life Sciences University of Eloued Eloued Algeria; ^3^ The Olive tree Institute of Sousse Sousse Tunisia; ^4^ The Olive tree Institute of Sfax Sfax Tunisia

**Keywords:** Chemlal, oil quality, polyphenol, Sigoise, tocopherol

## Abstract

In the present work, we are assessing the geographical origin and cultivar‐related changes on olive oil quality and composition in East Algeria. Fruits from the main local olive varieties (Sigoise and Chemlal) were harvested in autumn 2019 growing season from three different geographic areas: semiarid (Setif), arid (Batna), and Saharan (Eloued). Obtained results showed that Chemlal and Sigoise olive oils from Eloued area were the most enriched in tocopherols and phenolic contents, respectively. Sigoise olive oil from Batna area showed the highest values of pigments (carotenoids, chlorophylls). Identified fatty acids using gas chromatography (GC) coupled with mass spectrometry (MS) indicated that Chemlal cultivar olive oil from Batna was the most enriched in saturated fatty acid. However, higher levels of monounsaturated fatty acid were recorded for olive from Eloued and Setif areas. Sigoise cultivar oil displayed higher contents of unsaturated fatty acid in Batna, but higher levels of polyunsaturated fatty acid from Eloued location. These outcomes highlighted an actual impact of geographical location on each cultivar olive oil chemical proprieties. According to these data and relative to the International Olive Council (IOC) standards, all the analyzed olive oils could be categorized as extra virgin olive oils. Overall, statistical analysis showed that physicochemical parameters were influenced by the cultivar, the region of collection, as well as the interactions between them.

## Graphical abstract

Olive oil yield and chemical composition were significantly modulated by cultivar and geographic origin. Olive oil from Sigoise cultivar grown in the most arid region (Eloued) exhibited the best oil quality wealthy in essential fatty acids, antioxidants, and vitamin, but it behaves the lowest yield.

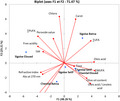

**Practical Applications**

This is a first report on bioclimatic and variety‐induced changes on Algerian olive oil. Here we found that olive oil from Sigoise cultivar grown in Eloued exhibited the best oil quality wealthy in essential fatty acids, antioxidants, and vitamins, but unfortunately it behaves the lowest yield. To produce olive oil of high yield with the best healthy quality, we suggest to farmers to blend the oils after extraction according to the following mixing combinations: oil Sigoise Eloued x Sigoise Setif.

## 1 INTRODUCTION

Olive growing in Algeria covers an area of 432.961 ha producing about 3.30% of world oil production (Hadj et al., 2018). The favorable climate and the ancestral olive growing traditions constitute a competitive advantage for the development of the olive sector and can contribute to the self‐sufficiency in vegetable oils. According to International Olive Council IOC and Newsletter Marché Oléicole (2017), Algeria produces 80,000 tons of olive oil and occupies ninth place at the global level, while this production is mainly dedicated for local consumption.

Currently, the olive tree is cultivated throughout the national territory, ranging from humid areas to arid and Saharan areas. The geographical distribution shows many important olive growing areas; especially, the coastal areas of the country are found to be favorable for the development of the olive tree. The coastal olive growing areas occupied more than 471,000 hectares (National Observatory of Agricultural and Agri‐food Sectors, 2016).

The majority of olive growing areas are located in mountainous regions and hills (Khoumeri, 2009), as well as in the western plains of the country (Mascara, Sig, Relizane, etc.) and in valleys such as Soummam and in the southern area between Biskra and Eloued delegations. Today, these areas of cultivation have increased significantly by the establishment of a national program for the development of intensive olive cultivation in the steppe areas, pre‐Saharan and Saharan.

The vast surface area of Algeria and the distinction of its bioclimatic stages associated with a diversity of cultivated varieties are factors which can however affect the quality of produced olive oil. There are many olive tree cultivars in Algeria; 36 cultivars are homologated by ITAFV (2006), where the most important cultivar of olive is Chemlal occupying 40% of the Algerian olive orchard, being cultivated for olive oil extraction. It has a high productivity and little alternating production season. It was rustic and late, self‐sterile, and always associated with other varieties that ensure its pollination as the varieties Sigoise or Azeradj. Chemlal cultivar is too often mistakenly confused with “Chemlali” Tunisian cultivar.

The Sigoise cultivar growing in the plain at Mascara region occupies 25% of the Algerian olive orchard; it has a dual purpose (olive oil and table olives). It is a seasonal cultivar, tolerant to salt water, moderately resistant to cold and drought. It is characterized by an early flowering with a medium intensity, low fruit set rate (0.70%), an average pulp‐core ratio (6.44%), the pulp is easily detached from the core, and the productivity is medium and alternate. It is a cultivar in extension on all the national territory and characterized as a good pollinator of Chemlal.

Currently, little work is dedicated to a comparative analysis of olive quality in Algeria and especially in the eastern region where new olive tree plantations are expanding. In the present work, we have undertaken a chemical characterisation of olive oil from two most common varieties in Algeria (Chemlal and Sigoise) harvested in three regions (Eloued, Batna, and north of Setif). The potential effects of cultivar and/or location were analyzed in terms of physicochemical properties of extracted oils, using a combination of colorimetric and chromatographic techniques.

## 2 MATERIAL AND METHODS

### 2.1 Experimental sites

This study was implemented in three of the most important olive growing regions of Algeria: Setif, Batna, and Eloued from the north, center, and south of Algeria, respectively. The geographical and climate proprieties of olive fruits collection sites are given in Table 1.

**TABLE 1:** Geographical and climate proprieties of olive fruits collection sites

	Bioclimatic stage	GPS coordinates	sea level	Annual mean temperature	Annual mean rainfall	Average relative humidity
Setif	semiarid	36°23'01.3"N 4°59'07.5"E	644 m	13.5°C	583 mm	77%
Batna	arid	35°18'33.5"N 5°22'08.5"E	468 m	22.4°C	195.9 mm	46%
Eloued	Saharan	33°21'22.1"N 6°47'42.1"E	87 m	25.6°C	65 mm	30%

### 2.2 Olive fruit and oil sampling

**FIGURE 1:**
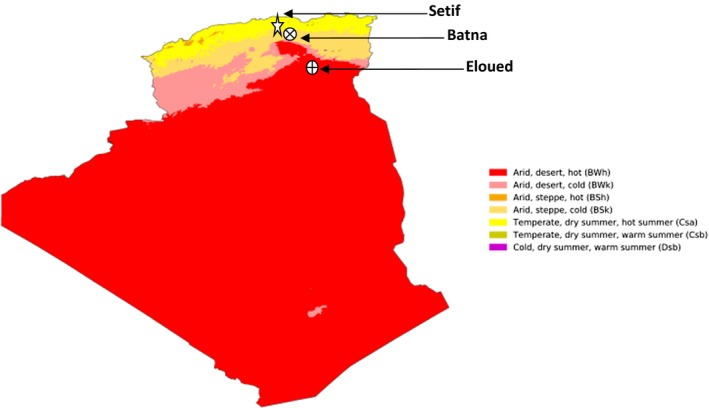
Collection sites of olive fruits as indicated in the Algeria map of Köppen–Geiger climate classification (Beck et al., 2018)

Olive fruit of the two predominate olive varieties in Algeria Chemlal and Sigoise were hand‐harvested from three different regions of Algeria as indicated in Figure 1. Olive fruits were collected manually from all the foliage during November 2019, with a maturity index (MI) ranging between 4 and 6 on a scale of 0 to 7. Hundred olives were randomly chosen from each collected olive sample (1 kg). Then MI was calculated by evaluating their skin and pulp colors. The MI was calculated as the mean of three values obtained for three olive samples collected in the same day from three different olive trees. For each cultivar, samples were randomly collected from 10 different olive trees in the field of each region. Harvested fruits were immediately conserved at 4°C until use.

### 2.3 Olive oil extraction

For each olive sample, fruits were processed and olive oil was obtained using an Abencor system (Commercial Abengoa SA, Sevilla, Spain). About 3–4 kg of olives from Chemlal and Sigoise cultivars from each location was cleaned from leaves, crushed with a hammer crusher, and the paste mixed at a temperature of about 27°C for 30 min, centrifuged in a decanter (1507 *g* over 3 min) without addition of warm water, transferred into dark glass bottles, and then stored at 4°C until analysis.

### 2.4 Determination of physicochemical parameters of oil samples

#### 2.4.1 Quality indices

Determination of free fatty acids, peroxide value (PV), and specific wavelength absorbance at 232 nm and 270 nm (K232 and K270) was carried out, following the analytical methods described in Commission Delegated Regulation (EU) (2016). Free fatty acids, expressed as % of oleic acid, were determined by titration of a mixture of oil sample (5 g) dissolved in ethanol (90 ml) with ethanolic solution of potassium hydroxide (0.1 N). Phenolphthalein was used as indicator. PV, expressed in milliequivalents of active oxygen per kilogram of oil (meq O_2_/kg), was measured in the following procedure: olive oil sample (1 g) was dissolved in a solution of chloroform‐acetic acid (10 ml), then the mixture was left to react with 1 ml of saturated solution of potassium iodide in darkness. The liberated iodine by the peroxides was titrated with standardized sodium thiosulphate solution using starch as indicator. K232 and K270 were calculated from absorption at 232 and 270 nm, respectively, with a UV spectrophotometer (SPECUVIS1; UV‐Visible), using a 1% solution of olive oil in cyclohexane (1 g/100 ml) and a path length of 1 cm.

#### 2.4.2 Refractive index

The refractive index or index of refraction (RI) is a ratio of the speed of light in a vacuum relative to that speed through a given medium (Hadj‐Taieb et al., 2012). This was evaluated using a refractometer apparatus (Waters, Milford, MA).

RI=velocity of light in a vacuum/velocity of light in medium

#### 2.4.3 Determination of total polyphenols

Phenolic compounds were isolated by double extraction of a solution of oil in methanol/Tween 20 mixture (2% v/w). The Folin–Ciocalteu reagent was added to a suitable aliquot of the extracts, and the absorption of the solution at 765 nm was measured (Singleton & Rossi, 1965). Values were given in milligram of hydroxytyrosol per kilogram of oil. Total phenols were expressed as gallic acid equivalents (GAE), using a calibration curve of a freshly prepared gallic acid solution.

#### 2.4.4 Pigments composition

Chlorophyll and carotenoid compounds as mg/kg of oil were determined at 670 and 470 nm in cyclohexane using the specific extinction values, by the method described by Wolff (1968):

$$\text{Chlorophyll}\;\left(\text{ppm}\right)=\frac{A_{670}‐\left(A_{630}+A_{710}\right)/2}{0.1086\times W}$$

where A is the absorbance and W is the spectrophotometer cell thickness (1 cm).

$$\text{Carotenoids}\;\left(\text{ppm}\right)=\frac{E_{0}\times 7.5}{\;A470\times 2\times 10,000}$$

*A*_470_: absorbance of the sample at 470 nm
*E*_0_: specific extinction = 2000.

### 2.5 Chromatographic analysis

#### 2.5.1 Fatty acids composition

Fatty acids were transformed into methyl esters using potassium hydroxide in methanol, according to International Olive Council (IOC) (International Olive Council & IOC/T.20/Doc. Nº 24, 2001), and analyzed by gas chromatography (GLC) using an HP 5890 chromatograph (Hewlett‐Packard, Palo Alto, CA, USA) with an FID detector. A BPX70 fused silica capillary column (50 m, 0.25 µm film; SGE, Incorporated, Austin, TX, USA) was used. The temperature was programmed between 160°C and 230°C at 2°C/min, and 0.5 µl samples were run with hydrogen as the carrier gas. The injection was carried out in split mode. Standard fatty acid methyl esters (FAME) from Sigma‐Aldrich Co. (St. Louis, MO, USA) were used for identification purpose. Fatty acids were identified by comparing retention times with standard compounds. Ten fatty acids were considered in this study. These were palmitic acid (16:0), palmitoleic acid (16:1n 7), margaric acid (C17:0), margaroleic acid (C17: 1n 8), stearic acid (18:0), oleic acid (18:1n 9), linoleic acid (18:2n 6), linolenic acid (18:3n 3), arachidic acid (20:0), and gondoic acid (C20:1n 9). Acids were expressed as percentages of FAMEs.

#### 2.5.2 Determination of tocopherols

Tocopherol analysis was determined according to AOCS method, official method CE8‐89. Olive oil (1 g) was weighed into a 10 ml volumetric flask and made up to volume with hexane. The tocopherol content was determined by HPLC, with hexane: isopropanol (99.5:0.5) as the mobile phase, with a flow rate of 1 ml/min. A silica column (25 cm 4 mm) granulometry 5µm was used. The temperature of the column was set to 25°C. The injection volume was 20 µl. The results were expressed in mg/kg.

### 2.6 Statistical analysis

Results were expressed as mean ± SD. The whole analysis was carried out with Minitab 17. Significant differences for comparison between olive cultivars and locations were determined by ANOVA, followed by the Fisher's LSD post hoc test for multiple comparisons with statistical significance of *p* < .05.

Principal component analysis (PCA), based on Pearson's product moment correlation at *p* < .05, was performed separately on physicochemical parameters. The analysis was performed using the XLSTAT software, Version 2009.4.03 (Addinsoft).

## 3 RESULTS AND DISCUSSION

### 3.1 Olive oil yield

**FIGURE 2:**
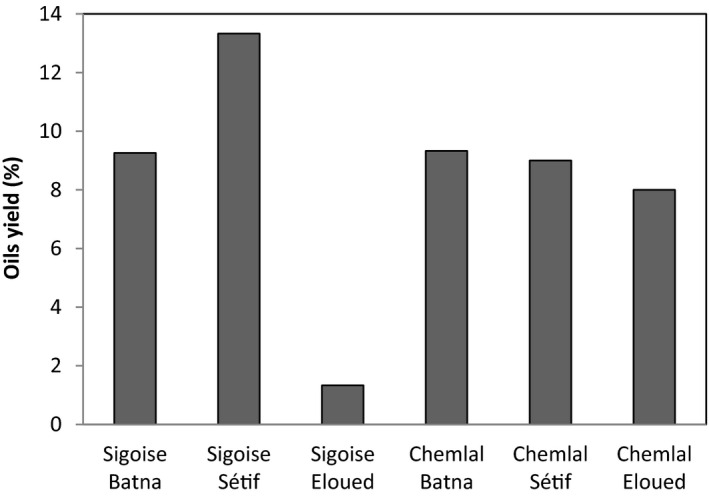
Olive oil yields of two varieties: Sigoise and Chemlal according to the geographical sites. Data are the mean of three‐repetition ± SD

The oil yield is one of the most important parameters to be determined since the main purpose of olive cultivation is the production of oil (Acila et al., 2017). On the other hand, oil yield is not a criterion for determining oil quality, but it is mainly a criterion to be considered in varietal selection (Abaza et al., 2002). The results of the oil content expressed as a percentage of fresh matter are presented as follows:

According to Figure 2, the oil yields expressed as a percentage of fresh matter vary from 1.33% to 13.33% in most samples. For Sigoise cultivar, the highest yield was noted in the Setif region (13.33%); however, the lowest one was observed for this cultivar when collected in the Eloued region at 1.33% (Figure 2).

Here, the severe oil yield drop of Sigoise could be due to the nature of the desert region Eloued which is characterized by high temperature where the olive trees are subjected to thermal stress during the fruit filling phase, leading to a reduction in oil yield (Acila et al., 2017). Besides, production alternation has characterized the Sigoise cultivar from the Eloued region; it is highly dependent on endogenous expression and environmental conditions and their interactions (Lavee, 2007; Toplu, Önder, et al., 2009; Toplu, Yildiz, et al., 2009). This attribute appears when certain cultivars tend to produce higher yields in one year followed by a decrease in yields the following year (Pearce & Doberšek‐Urbanc, 1967). Alternation of production can be one of the main limiting factors in olive production. This phenomenon is attributed to competition for assimilation during bud differentiation, inflorescence growth, fruit set, fruit growth, and vegetative growth (Cuevas et al., 2009; Proietti, 2003). Cultural practices applied to orchards, including pruning, fertilization, and irrigation, contribute to reducing their intensity (Lavee, 1997; Vossen & Kicenik Devarenne, 2007). This low yield can also be explained by the relationship between the degree of fruit ripening of this cultivar in this geographical area and oil yield. Ait et al. (2001) noted a strong correlation between maturity index and oil content for the Moroccan Picholine cultivar.

According to Avidon et al. (1997), the oil yield of ripe olives varies from 5% to 35% oil on fresh material. Our results are a bit inferior to the work obtained by Douzane and Bellal (2004) during the 1996/1997 season on six cultivated varieties from the Bejaia region (Algeria) where the oil yield varies from 8% to 15.76%.

In addition, the Chemlal cultivar destined for oil presented a stable yield between 8% and 9% and is not affected by the geographical location. The analysis of all the results showed that regarding adaptation and production criteria, the Chemlal cultivar is more adapted or well tolerated to the different bioclimatic conditions analyzed, although the Sigoise cultivar is the most productive in arid and semiarid areas, but less productive in the Saharan zone (Sidhoum & Gaouar, 2013).

### 3.2 Free fatty acid contents

Free fatty acid content is a factor that provides an indication of the alteration of the oil by hydrolysis. The results recorded for the analyzed samples are shown in Table 2.

**TABLE 2:** Table of physicochemical parameters of olive oil from two varieties: Sigoise and Chemlal depending on the geographical locations. Means sharing at least one letter are not significantly different according to Fisher's LSD post hoc test (*p* < .05)

Cultivars	Location	Free fatty acids %	Peroxide value (meq O_2_/kg)	Total Polyphenols (ppm)	Chlorophyll mg/kg	Total Carotenoids mg/kg	K232	K270	Refractive index
Sigoise	Setif	0.22 ± 0.01^b^	29.10 ± 0.25^a^	617.91 ± 57.25^b^	0.59 ± 0.16^d^	12.06 ± 0.02^bc^	1.920 ± 0.106^a^	0.139 ± 0.007^ab^	1.4703 ± 0.0003^a^
	Batna	0.20 ± 0.02^b^	19.87 ± 0.12^c^	382.87 ± 0.21^c^	5.56 ± 0.12^a^	24.30 ± 1.18^a^	1.654 ± 0.036^a^	0.107 ± 0.012^b^	1.4678 ± 0.0001^d^
	Eloued	0.30 ± 0.01^a^	26.35 ± 0.25^b^	891.53 ± 53.96^a^	4.10 ± 0.07^b^	12.97 ± 0.06^b^	2.073 ± 0.029^a^	0.208 ± 0.006^a^	1.4701 ± 0.0002^a^
Chemlal	Setif	0.20 ± 0.01^b^	13.60 ± 0.75^e^	692.03 ± 20.71^b^	0.88 ± 0.12^cd^	13.14 ± 0.02^b^	1.842 ± 0.173^a^	0.139 ± 0.025^ab^	1.4681 ± 0.0000^d^
	Batna	0.19 ± 0.00^b^	9.52 ± 0.17^f^	324.95 ± 14.79^c^	0.68 ± 0.13^cd^	9.62 ± 1.81^c^	2.060 ± 0.010^a^	0.150 ± 0.012^ab^	1.4690 ± 0.0001^b^
	Eloued	0.22 ± 0.02^b^	17.37 ± 0.22^d^	394.12 ± 30.37^c^	1.02 ± 0.04^c^	10.73 ± 0.97^bc^	1.929 ± 0.262^a^	0.188 ± 0.041^a^	1.4686 ± 0.0001^c^
*p* Values of main effects	Location	.004	.000	.000	.000	.004	.561	.042	.001
	Cultivar	.014	.000	.002	.000	.001	.600	.705	.000
*p* Values the interactions	Loc. × Cultivar	.051	.000	.001	.000	.000	.169	.415	.000

The results recorded in Table 2 showed that the values obtained meet the standards of the IOC (International Olive Council, 2019), which recommends a free acidity lesser or equal to 0.80 g of oleic acid per 100 g of oil. Obtained values were between a minimum of 0.19 g registered in the Chemlal cultivar of Batna and a maximum of 0.30 g in the Sigoise cultivar of Eloued. These values were close to those of the Tunisian cultivar oils (*Olea europaea* L. cv. Zelmati) for which the free acidity ranged between 0.13 and 0.33 g of oleic acid (Ben Rouina et al., 2020), but lower than those recorded in the Chetoui cultivar from 0.30 to 0.90 g of oleic acid per 100 g of oil.

The acidity values recorded in Table 2 indicated that the acidity levels of olive oil in the different studied regions are in accordance with the standards established by International Olive Council (2019), which classify them in the category of extra virgin oils. The results of the analysis of variance showed that there was a highest significant effect with a *p*‐value of around .004 for the factor location compared to the *p*‐value of .014 for the cultivar. Conversely, there was no significant effect for the interaction factor: cultivar–region, where *p*‐value = .051.

### 3.3 The peroxide value

The PV is related to the harvest, storage, and extraction method. It reflects the degree of oxidation of oils, accelerated by the presence of oxygen, temperature, and certain catalysts; these factors act on the double bonds of unsaturated fatty acids to form peroxides and hydroperoxides (Cimato, 1990). The results of the PV were shown in Table 2. According to the standards of the IOC 2019, a PV must be less than or equal to 20 meq O_2_/kg. Indeed, the PV for samples of olive oil of the Sigoise cultivar from the Setif region (29.10 meq O_2_/kg) and Eloued (26.35 meq O_2_/kg) marks a slight exceedance of the standard of International Olive Council (2019) (Table 2). These values of the PV indicate a strong oxidation of the oils; this could be due to several conditions during the stages preceding the extraction of the oil (harvesting), and inadequate or prolonged storage is also one of the causes of increase of this parameter (Meftah et al., 2014; Tanouti et al., 2011). Nevertheless, the remaining samples showed consistent PVs ranging from 9.35 meq O_2_/kg to 20 meq O_2_/kg within the limit determined by International Olive Council (2019).

The PVs obtained were lower than those recorded by Douzane and Bellal (2004) for the oil of the varieties (Azeradj, Chemlal, Limli, Takesrit, Aghenfas, and Grosse du Hamma) of Bejaïa, and similar to those mentioned by Özcanb et al. (2007) of the oil from five varieties of olive tree (Ayvalık, Gemlik, Kilis, Tirilye, and Uslu) where PVs range from 15.3 to 22.5 meq O_2_/kg.

The results of the analysis of variance at one classification criterion showed that there are very highly significant differences for the two factors: oil PV and cultivar (*p* = .00), and there were also very highly significant differences for the two factors: PV and region.

### 3.4 Determination of total polyphenol contents

The polyphenol content in the oil sample changes during the extraction process. Obtained results, as presented in Table 2, showed high accumulation of polyphenols in different extracted oils. According to our reported data in Table 2, it was noticeable that olive oil from Sigoise Eloued cultivar contains the highest amount of polyphenol compounds (891.53 ± 53.96 mg/kg), while the lowest contents were recorded for oil of Chemlal Batna (324.95 ± 14.79).

Based on total polyphenol levels, the olive oil samples can be divided into three categories: (i) oils with low polyphenol contents, <400 mg/kg corresponding to the oils produced from the varieties Sigoise and Chemlal of Batna and then Chemlal of Eloued; (ii) oils with average polyphenol contents between 400 and 800 mg/kg and correspond to the oils of Setif varieties Chemlal and Sigoise; and (iii) oils with high polyphenol contents, higher than 800 mg/kg which was the case of Sigoise from Eloued (Table 2). These are olive oils from the Sigoise Eloued cultivar. Variations in the observed polyphenol levels may be due to the different degree of maturity of the olives before crushing (early harvest of the olives) but also depends on the cultivar and the geographical area as reported by García et al. (2003).

The results obtained by Guerfel et al. (2012) showed a total polyphenol content in Chemlali oil reaching 890 ± 9.50 mg/kg, which is similar to results recorded for the Sigoise cultivar of Eloued (Table 2). These polyphenol levels were also higher than those obtained by Baccouri et al. (2008) for Chetoui cultivar olive oil from 363.90 to 567.60 mg/kg. According to Table 2, it could be stated that oil polyphenol levels were more affected by geographical location (*p* =.00) rather than olive tree cultivar (*p* =.002). For instance, Sigoise cultivar oil polyphenol contents were at least 2‐fold higher for Setif and Eloued locations compared to Batna ones (Table 2). A highly significant effect was recorded between varieties and geographical regions (*p* =.001) in polyphenol content. Karabagias et al. (2013) studying 47 monovarietal olive trees collected from four Western Greek islands showed that when numerous of these physicochemical parameters were combined with chemometric, the olive oil diversity was related to the geographical origin.

### 3.5 Pigment contents

#### 3.5.1 Chlorophyll contents

The results of the average chlorophyll contents of the two olive oil cultivars expressed in (mg/kg) in different studied regions are summarized in Table 2. Oils from the Sigoise cultivar from Batna and Eloued regions were the richest in chlorophyll, reaching 5.60 and 4.10 mg/kg, respectively (Table 2). The chlorophyll contents of the other samples were strictly inferior to 1.10 mg/kg (Table 2). These fluctuations in chlorophyll levels may be related to the degree of maturity of the olives (Boulfane et al., 2015). According to Benrachou et al. (2010), this decrease was due to the degradation of chlorophyll under the action of chlorophyllases into pheophytins that give the oil its yellow color. Studies have shown that these chlorophyll pigments, the majority of which are found in olives, degrade rapidly during olive ripening, which explains our results. However, for Sigoise Batna and Sigoise Eloued, we found high average values of 5.56 and 4.10 mg/kg, suggesting possible contamination of the olive by the leaves. Indeed, (Ben Tekaya & Hassouna, 2007) pointed out the interest of producing olive oils from ripe olives and proceeding to leaf removal during oil extraction.

Our chlorophyll content values are higher than those reported by Zegane et al. (2015) of Algerian olive oil ranging from 0.84 to 2.89 mg/kg. Similar chlorophyll contents were measured for extra virgin olive oils of the Tunisian (Chetoui) cultivar (Ben Tekaya & Hassouna, 2007).

The results of the analysis of variance showed that there were very highly significant differences (*p*‐value = .00) either for the two factors: chlorophyll rate of the oil and the geographical origin or the chlorophyll rate and the cultivar, as well as for the interaction factor: cultivar–region.

#### 3.5.2 Carotenoid contents

Carotenoids are natural chemical substances involved in the oxidation mechanisms of oils. Their presence in sufficient quantities in the oil allows to delay the photooxidation and to preserve the quality parameters of the oil during storage (Lazzer et al., 2006). The values of carotenoid contents of the two olive oil cultivars for different studied regions were illustrated in Table 2. With the exception of the Batna Sigoise cultivar, which had the highest average carotenoid contents of 24.3 mg/kg, the averages of the other samples recorded convergent values at the carotenoid contents level of 9.62 to 13.14 mg/kg (Table 2). Our results were relatively higher compared to those obtained for other Algerian olive varieties with carotenoid contents ranging from 2.56 to 16.35 mg/kg (Hadj et al., 2018). On the other hand, these results were close to the work reported by Dabbou et al. (2010) for Tunisian olive oil where carotenoid levels fluctuate between 6.58 and 20.03 mg/kg. However, statistical analysis revealed a very significant difference in terms of geographical site (*p* = .004) and also showed that the oil from the Batna region has higher carotenoid value (33.91 mg/kg) than that from the Setif and Eloued regions (25.19 mg/kg, 23.71 mg/kg), respectively. However, there is higher significant difference for the cultivar factor (*p* = .001), and the Sigoise cultivar contains a higher carotenoid content (49.46 mg/kg) than the Chemlal cultivar (33.53 mg/kg). The higher significant difference for the interaction factors: cultivar x region (*p* = .00) indicated that carotenoid content was concomitantly regulated by cultivar and geographical location (Table 2).

### 3.6 Determination of the specific extinction coefficient

The values for the determination of the UV absorption coefficients (K232, K270) of conjugated bonds provide information on the presence or absence of precursors or on the onset of oxidation and thus on the prediction of the oil stability (Hadj et al., 2018). The results were presented in Table 2

It should be noted that the values of the K232 absorption coefficients of the oils of all assayed varieties do not exceed the limit established by International Olive Council (2019), which was 2.5. The highest values recorded in the cultivar Sigoise oil from Setif and Eloued, in addition to the absorbance at 270 nm which was ≤0.22, (∆*K* ≤ 0.01) were in respect with the thresholds set for extra virgin olive oil (Table 2).

According to Tanouti et al. (2011), these values would be related to several factors such as late harvesting of olives, excessive exposure of the olives, and the extracted oil to oxygen from the air and light, or even to heating of the paste during crushing. In fact, with the results of the PV of Sigoise Setif and Sigoise Eloued (29.10 and 26.35 meq O_2_/kg of oil), similar results were also reported by Boulfane et al. (2015) who noted that these factors had a significant impact on absorbance.

The specific extinction of oil at 232 nm and 270 nm reflects its oxidation state. The stronger its extinction at 232 nm, the more peroxidized it is. Similarly, the higher the extinction at 270 nm was, the richer the oil was in secondary oxidation products which reflects its low storability (Wolff, 1968). The recorded specific extinction coefficients were close to those obtained by Ruiz‐Dominguez et al. (2013) on olive oil varieties of the Valencia region (Spain) where the K232 fluctuated from 1.17 to 2.21 nm and the K270 coefficient between 0.08 and 0.21 nm. Virgin olive oil of some varieties in Argentina presented values ranging from 1.61 to 1.93 nm for K232 and between 0.07 and 0.15 nm for K270 (Torres & Maestri, 2006).

The values of K232 showed that there was no significant difference with the factors of cultivar, geographic region, and for the interaction factor: cultivar–region, where *p*‐value ˃ .05. However, the values of K270 showed that there were significant effects with the geographical factor (*p* = .042) and no significant effects for the cultivar factor and the interaction factor also: cultivar–region.

### 3.7 Determination of the RI

The results of the refractive indices of the different olive oil samples are shown in Table 2. The average RI of the analyzed samples ranged from 1.4678 to 1.4703. All these values meet the standard established by CODEX (2009), suggesting to categorize these extracted oils as pure. Obtained values were close to those reported for Tunisian extra virgin olive oil (Ben Tekaya & Hassouna, 2005) and for five Turkish olive oils (Özcanb et al., 2007) ranging from 1.4690 to 1.4700 and 1.4670 to 1.4690, respectively.

The RI of oils depends on the structure of fatty acids and the degree of esterification; it increases with the number of carbon atoms, the degree of unsaturation, and conjugation and takes higher values for monoglycerides than for triglycerides (Gunstone, 2011). Statistically, there were a very significant differences related to geographical location, olive tree cultivar, and the interaction factor: cultivar x location (*p* ≤ .000).

### 3.8 Fatty acid composition of olive oil

The analysis of methyl esters of total fatty acids, by gas chromatography, allowed us to identify several fatty acids in each analyzed olive oil. The individual fatty acid compositions of the analyzed oils were given in Table 3. The data showed that the analyzed oils have average values of fatty acid content that meet the commercial standards established by International Olive Council (2019), for extra virgin olive oil, except for the average linolenic acid of the Sigoise Eloued cultivar of 1.34% which slightly exceeds the upper limit tolerated which is 1%. This value was also reported by Hadj et al. (2018), in a study on the same cultivar Sigoise (1.29% linolenic acid).

**TABLE 3:** Total fatty acid composition of olive oils from two varieties: Sigoise, Chemlal depending on the geographical locations. Means sharing at least one letter are not significantly different according to Fisher's LSD post hoc test (*p* < .05)

Areas Fatty acids	*Sigoise*			*Chemlal*			IOC Standard 2019
	Setif	Batna	Eloued	Setif	Batna	Eloued	
C16:0	16.91 ± 0.04^c^	12.71 ± 0.01^f^	17.91 ± 0.03^b^	16.58 ± 0.04^d^	19.73 ± 0.14^a^	15.02 ± 0.04^e^	**7.5−20**
C16:1	1.35 ± 0.16^b^	0.51 ± 0.07^d^	1.05 ± 0.03^c^	1.54 ± 0.09^b^	2.46 ± 0.06^a^	0.66 ± 0.02^d^	0.3−3.5
C17:0	0.01 ± 0.00^d^	0.02 ± 0.00^cd^	0.04 ± 0.01^ab^	0.03 ± 0.01^bc^	0.01 ± 0.00^d^	0.05 ± 0.01^a^	<0.5
C17:1	0.06 ± 0.01^b^	0.06 ± 0.00^b^	0.08 ± 0.01^a^	0.06 ± 0.00^b^	0.06 ± 0.00^b^	0.07 ± 0.01^ab^	<0.6
C18:0	1.87 ± 0.06^d^	4.36 ± 0.01^a^	1.94 ± 0.03^d^	2.22 ± 0.03^c^	1.64 ± 0.01^e^	2.80 ± 0.02^b^	0.5−5
C18:1	66.64 ± 0.30^c^	67.66 ± 0.13^b^	60.45 ± 0.07^e^	67.51 ± 0.04^b^	62.62 ± 0.13^d^	69.47 ± 0.03^a^	55−83
C18:2	12.45 ± 0.07^c^	13.74 ± 0.10^b^	16.20 ± 0.03^a^	11.37 ± 0.06^d^	13.01 ± 0.15^b^	10.96 ± 0.04^e^	3.5−21
C18:3	0.44 ± 0.01^d^	0.70 ± 0.02^c^	1.34 ± 0.03^a^	0.50 ± 0.01^d^	0.65 ± 0.03^c^	0.81 ± 0.01^b^	<1
C20:0	0.16 ± 0.01^ab^	0.22 ± 0.08^ab^	0.07 ± 0.02^b^	0.24 ± 0.03^a^	0.08 ± 0.05^b^	0.26 ± 0.05^a^	<0.6
C20:1	0.11 ± 0.05^ab^	0.05 ± 0.02^b^	0.12 ± 0.01^ab^	0.15 ± 0.01^a^	0.11 ± 0.02^ab^	0.14 ± 0.01^a^	<0.4
(C18:1/C18:2)	5.35 ± 0.05^cd^	4.93 ± 0.04^a^	3.73 ± 0.01^e^	5.94 ± 0.03^bc^	4.82 ± 0.04^d^	6.34 ± 0.02^b^	−
∑SFA	18.94 ± 0.03^c^	17.30 ± 0.07^e^	19.95 ± 0.01^b^	19.06 ± 0.05^c^	21.45 ± 0.09^a^	18.11 ± 0.09^d^	−
∑UFA	81.04 ± 0.04^c^	82.71 ± 0.10^a^	79.22 ± 0.15^d^	81.12 ± 0.13^c^	78.90 ± 0.25^d^	82.09 ± 0.11^b^	−
∑MUFA	68.15 ± 0.10^d^	68.30 ± 0.20^c^	61.69 ± 0.10^f^	69.25 ± 0.06^b^	65.25 ± 0.08^e^	70.33 ± 0.05^a^	−
∑PUFA	12.89 ± 0.06^c^	14.44 ± 0.12^b^	17.54 ± 0.06^a^	11.87 ± 0.07^d^	13.65 ± 0.17^b^	11.76 ± 0.04^d^	−

In addition, Table 3 detailed the main fatty acids that make up these fats such as palmitic acid (C16:0), oleic acid (C18:1 ω9), and linoleic acid (C18:2 ω6). These levels of major fatty acids were also similar to those previously observed by other authors (Dabbou et al., 2010; Manai‐Djebali et al., 2012; Tanouti et al., 2011). There were minority fatty acids as well, with percentages below 5% such as stearic acid (C18:0), linolenic acid (C18:3 ω3), and palmitoleic acid (C16:1). Trace fats with percentages not exceeding 0.3% have been identified, such as heptadecanoic acid (C17:0), heptadecenoic acid (C17:1), arachidic acid (C20:0), and gadoleic acid (C20:1).

Oleic acid is the dominant fatty acid in the composition of various oil samples; it exceeds 69% in Chemlal Eloued (Table 3). While the other samples range from 60% to 67%, these rates are moderately classified in the range of extra virgin olive oils (55% to 83%).

The highest percentages of palmitic acid and palmitoleic acid are recorded in Chemlal and Batna oil with values of 19.73% and 2.46%, respectively (Table 3). The percentages of the other oils range from 12.71% to 17.91% for palmitic acid and from 0.51% to 1.54% for palmitoleic acid. Concerning the stearic acid average, it showed higher content in Batna oil (4.36%) compared to the different oils analyzed.

The analysis of the data revealed the richness of oil from Sigoise Eloued in linoleic acid (16.20%), linolenic acid (1.34%), and heptadecenoic acid (0.08%); these values are in average the highest in comparison to other results related to the study of these essential fatty acids. It is also interesting to note that the levels of essential fatty acids (linoleic (18:2, ω 6) and linolenic (18:3, ω3) contained in the different oils were sufficient to prevent a deficiency state in essential fatty acids in people using these oils as the main fat in their diet (Benrachou et al., 2010; Lapillonne et al., 2003).

The oleic acid/linoleic acid ratio is relatively higher than that of Chemlal Eloued oil (6.34). For oils from other olives, the average ratios range from 3.73 to 5.94. These results were highly variable for all the studied oils (Table 3). According to the study cited herein (Gutiérrez et al., 1999), there is an inversely proportional relationship between oleic acid and linoleic acid; with the formation of oleic acid, the enzyme oleate desaturase is active to transform oleic acid into linoleic acid during ripening.

**FIGURE 3:**
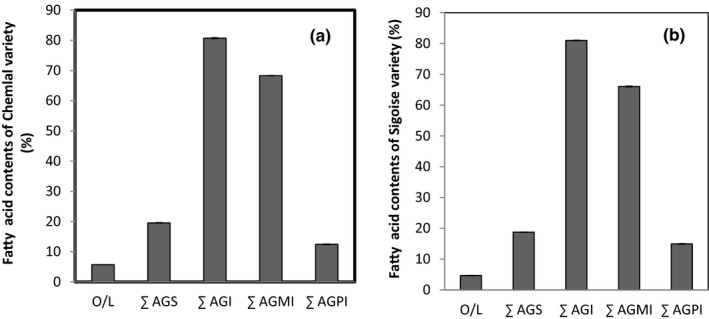
Fatty acid content groups of olive oils from the different geographical sites depending on two varieties: (a) Chemlal and (b) Sigoise. Data are the mean of three‐repetition ± SD

Another interesting factor to consider for distinguishing between oils was the proportion of the different classes of fatty acids (Table 3, Figure 3). In fact, the olive oil of Chemlal Batna stands out for its predominance in saturated fatty acids (21.45%), while the low content was recorded for Sigoise Batna (17.30). On the other hand, the oil from Sigoise Batna showed the highest level of unsaturated fatty acids (82.71%), and the lowest ones were recorded for Chemlal Batna (78.90%). As for the oil from Sigoise Eloued, it has the highest percentage value in polyunsaturated fatty acids of 17.54% and the highest base value in monounsaturated fatty acids of 61.69% compared to other oils (Table 3). Chemlal Eloued was rich in monounsaturated fats but showed low polyunsaturated fatty acid contents. To determine the variation of fatty acid group content from a cultivar point of view or from geographical origin contribution by calculating the sum of groups in each cultivar and each region of studied olive oil, the results were shown in Figure 3a and b.

The recorded results seem to follow the fluctuation of the ratio of the O/L sum between the two analyzed varieties, of which the Sigoise cultivar had a rather high ratio (5.70) than the Chemlal cultivar (4.67) (Figure 3). For the levels of saturated and polyunsaturated fatty acids, there was a slight superiority for the Chemlal cultivar; nevertheless, the most important average values of unsaturated and monounsaturated fatty acids were those corresponding to the Sigoise cultivar compared to that of the Chemlal cultivar (Figure 3). These values showed that the variation in the fatty acid content seems to be linked to the cultivar factor.

### 3.9 Total tocopherol contents

Tocopherols are known for their dual beneficial action. Firstly, they have the advantage of being a fat‐soluble vitamin (vitamin E), and secondly, they have a strong antioxidant activity (Burton & Ingold, 1986). The total tocopherol content in olive oils is highly variable (Boskou et al., 2006).

According to Sherwin (1976), alpha‐tocopherol alone accounts for 90% of all tocopherols; this form has the highest vitamin activity and is the most active. It opposes rancidity and polymerization of the oil, but there are also some beta and gamma tocopherols, while delta tocopherol is present only in trace amounts (Psomiadou et al., 2000; Schwartz et al., 2008).

The data showed variations in the levels of tocopherols found in the studied six olive oil samples, with values ranging from 94 to 345 mg/kg of oil (Table 4). Indeed, the highest tocopherols content was observed in the olive oil of the Chemlal cultivar from Eloued region, while the lowest value was recorded in the Sigoise cultivar from Setif region. Similar changes in tocopherol contents were reported for Italian (Lucci et al., 2020) and Greek olive oils (Psomiadou et al., 2000) with values ranging from 142 to 344 mg/kg and 98 to 370 mg/kg, respectively.

**TABLE 4:** Total tocopherol content (mg/kg) of olive oils from two varieties: Sigoise, Chemlal depending on the geographical areas. Means sharing at least one letter are not significantly different according to Fisher's LSD post hoc test (*p* < .05)

Areas	Sigoise			Chemlal		
	Setif	Batna	Eloued	Setif	Batna	Eloued
Total tocopherols	94.02^b^	199.03^a^	226.63^c^	115.13^b^	180.70^a^	345.65^d^

The highest level of total tocopherols among geographical areas was recorded in the most arid region of Eloued with a level of 345.65 mg/kg in Chemlal oil and 226.63 mg/kg in Sigoise one (Table 4). Many authors explain that the variability in total tocopherol content depends on several reasons such as cultivar, ripening stage, agroclimatic conditions, olive growing techniques, and storage conditions (Beltrán et al., 2005; Cunha et al., 2006; Psomiadou et al., 2000). Here, moving from the north (Setif) to the south (Eloued) was associated with increasing oil tocopherol content (Table 4). It was reported that increasing tocopherol content was related to tolerance of drought stress conditions in *Catharanthus roseus* (Abdul et al., 2007). Both olive tree cultivars showed similar total tocopherol change patterns which were rather affected by geographical factor than olive tree cultivar.

### 3.10 The principal component analysis

We also performed a PCA on the studied traits and varieties in order to show associations of individuals or links between variables. The numerical analysis was done using the XLStatPro v 7.5.2 software.

**FIGURE 4:**
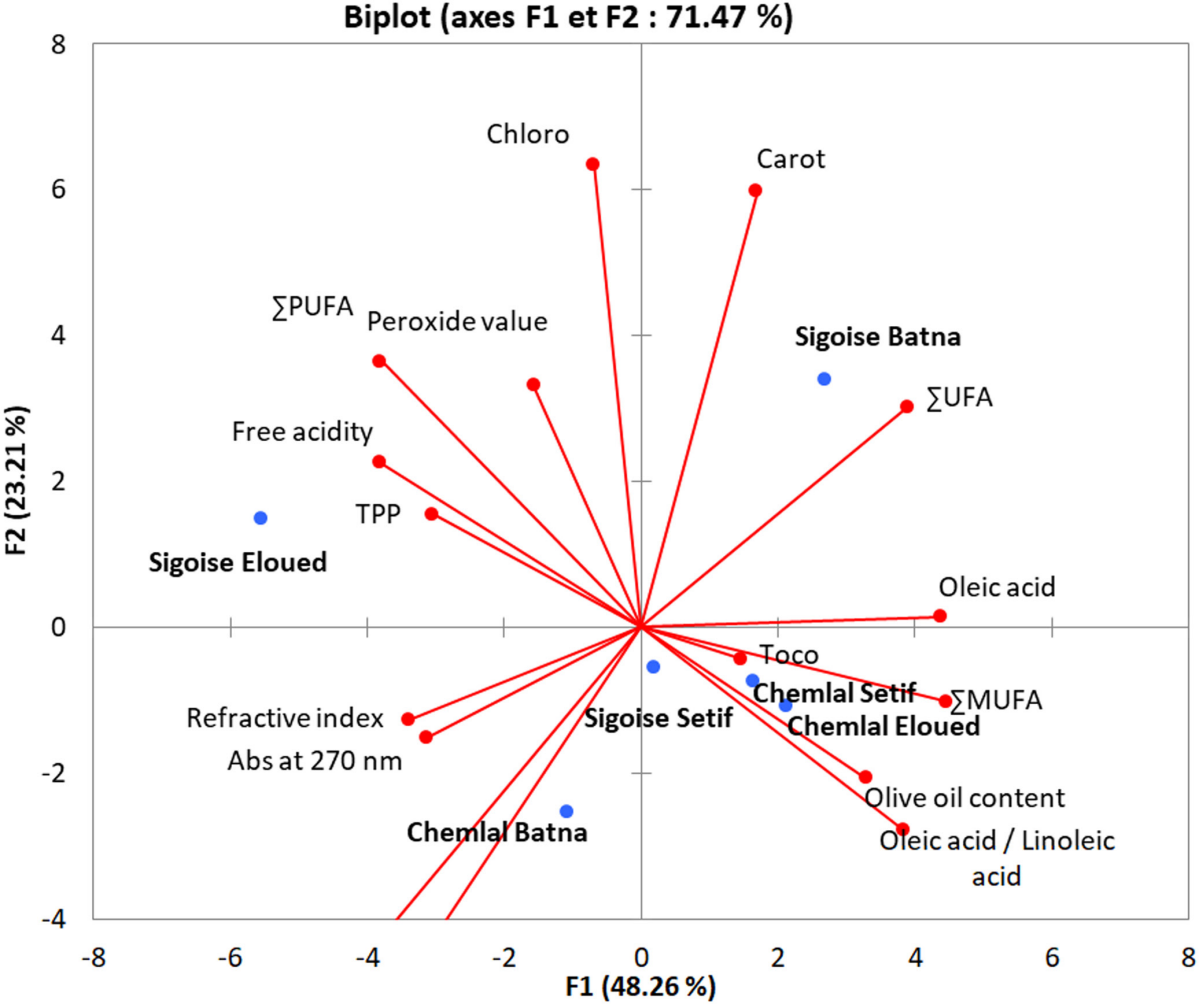
Principal component analysis of physicochemical parameters: projection of variables and cultivars on the factorial plane 1–2

The PCA performed on the variables of physicochemical parameters: olive oil content, free acidity, PV, RI, absorbance at 232 nm, absorbance at 270 nm, total phenols, chlorophylls, carotenoid, tocopherols, oleic acid, oleic acid / linoleic acid, the sum of (saturated fatty acids, unsaturated fatty acids, monounsaturated fatty acids, and polyunsaturated fatty acids), was presented in Figure 4.

The results of this analysis showed that the first two axes (1 and 2) alone explain the majority of the observed variability, that is, 71.47%. Thus, we will only represent the dispersion of the variables and the individuals in the PCA plane, generated by axes 1 and 2.

The first component is the most significant and explained 48.26% of the total variation. It was positively correlated to the variables related to the cultivar Sigoise of Batna region; total carotenoids, the sum of unsaturated fatty acid and Oleic acid. The F1 axis was also positively correltaed to the variables related to the varieties Chemlal and Sigoise of Setif region and Chemlal of Eloued for the parameters tocopherols, olive oil content, Oleic acid/Linoleic acid, the sum of monounsatured fatty acids. In the negative direction of this axis, we found the variables related to the cultivar Sigoise Eloued; free fatty acids, total polyphenols, the sum of polyunsaturated fatty acid, PV and chlorophylls. A negative correlation was also observed for the cultivar Chemlal Batna parameters; RI, the absorbance at 230 nm and at 270 nm, the sum of saturated fatty acid.

The second component accounted for 23.21% of the variation. It was positively correlated to the measured parameters of sigoise cultivars growing in Batna: total carotenoids, the sum of unsaturated fatty acid and Oleic acid, as well as to the variables of Sigoise Eloued: free fatty acids, total phenols, the sum of polyunsaturated fatty acid, PV and chlorophyll. F2 axis was negatively correlated to the varieties of Chemlal and Sigoise from Setif region and Chemlal of Eloued for the olive oil tocopherols content, Oleic acid/Linoleic acid, the sum of monounsaturated fatty acids, and those related to the cultivar Chemlal Batna for RI and the sum of saturated fatty acid (Figure 4).

According to Tables 4 and 5, a wide dispersion of the characteristics of the studied varieties appeared which translates the existence of a great inter‐varietal variability for many physicochemical parameters.

**TABLE 5:** Percentage of inertia and contribution of the physicochemical parameters to the different axes of the principal component analysis

Variables	Correlations between variables and axes	
Olive oil content	0.699	−0.306
Free fatty acids	−0.813	0.335
Peroxide value	−0.335	0.49
Refractive index	−0.726	−0.188
Absorbances at 232 nm	−0.774	−0.604
Absorbances at 270 nm	−0.673	−0.225
Total polyphenols	−0.65	0.231
Chlorophyll	−0.149	0.941
Carotenoids	0.363	0.889
Tocopherols	0.309	−0.063
Oleic acid	0.932	0.02
Oleic acid / Linoleic acid	0.82	−0.412
∑SFA	−0.648	−0.631
∑UFA	0.832	0.449
∑MUFA	0.948	−0.15
∑PUFA	−0.811	0.541
**Main components (axes)**	**Axis 1**	**Axis 2**
Eigen value	7.722	3.714
Variability (%)	48.261	23.213
Cumulative (%)	48.261	71.473

Contributions et cosinus carrés des individus (cultivars) sur le plan factoriel 1–2						
Observation	Coordinates		Contributions		square cosines	
	F1	F2	F1	F2	F1	F2
Sigoise Batna	2.682	3.407	15.527	52.082	0.353	0.569
Sigoise Setif	0.193	−0.536	0.081	1.292	0.004	0.033
Sigoise Eloued	−5.547	1.492	66.407	9.983	0.913	0.066
Chemlal Batna	−1.081	−2.539	2.522	28.927	0.088	0.488
Chemlal Setif	1.627	−0.741	5.711	2.464	0.402	0.084
Chemlal Eloued	2.126	−1.082	9.752	5.252	0.335	0.087

**FIGURE 5:**
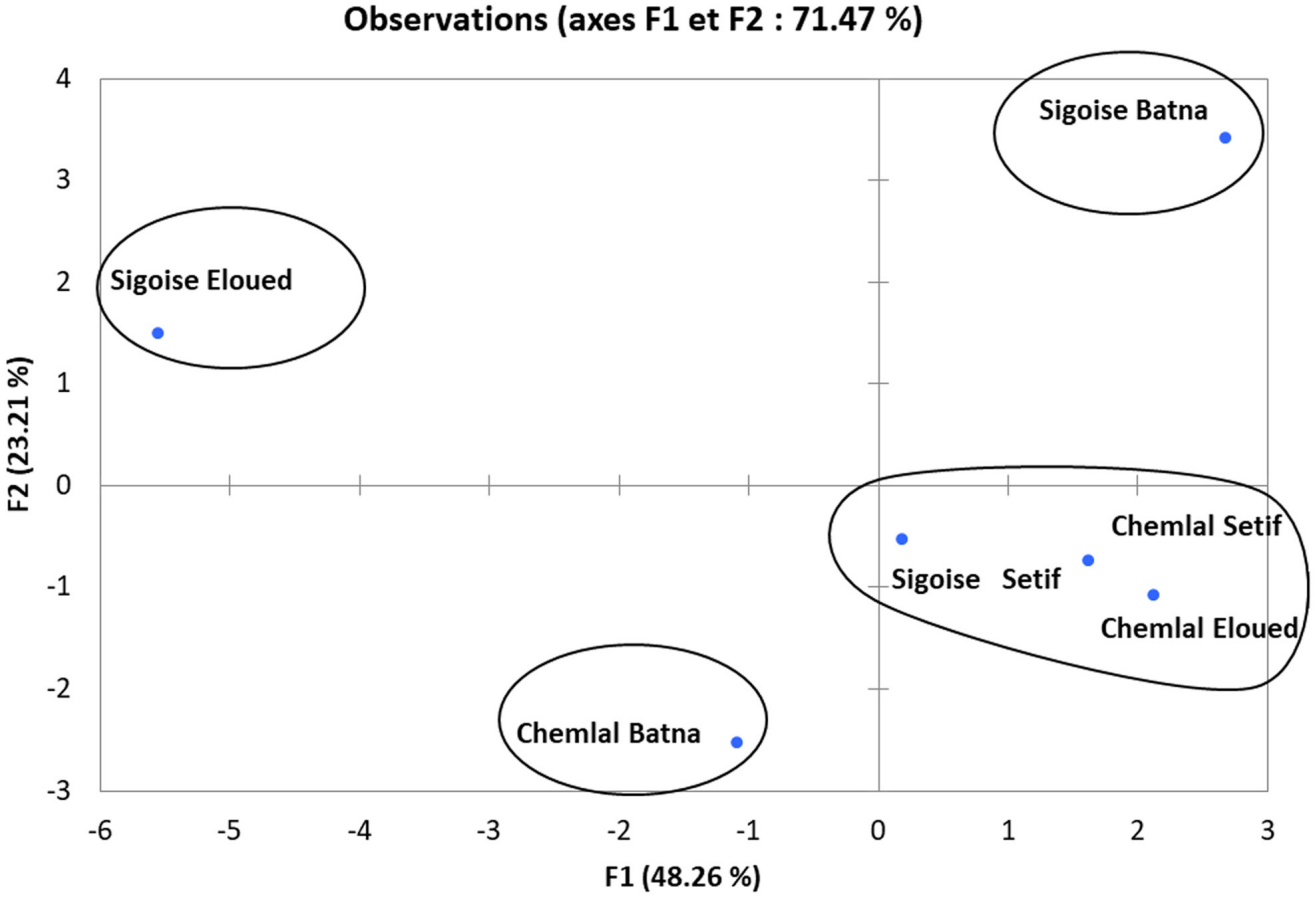
Representation of cultivars on the factorial plane 1–2

The projection of the two olive cultivars in the factorial plane (1–2) showed their diversity for the studied descriptors (Figure 5).

For the cloud of individuals projected on axis 1 and 2, these population varieties are divided into four groups:

‐ The first group: the cloud formed by these population varieties was destined toward the positive side of axes 1 and 2. It gathers the cultivar population Sigoise Batna.
‐ The second group: the cloud formed by these population varieties was intended toward the negative side of axis 1 and toward the positive side of axis 2. It includes the varieties populations Chemlal Eloued, Chemlal Setif, and Sigoise Setif.
‐ The third group: the cloud formed by these population varieties was intended to the positive side of axis 1 and to the negative side of axis 2. It includes the cultivar population Sigoise Eloued.
‐ The fourth group: the cloud formed by these population varieties was intended toward the negative side of axis 1 and toward the negative side of axis 2. It groups the cultivar population Chemlal Batna.

The analysis in principal components indicated that the varieties of olive tree of different studied regions also presented a wide diversity between the varieties in qualitative, chemical, or physical characteristics of the oil distinguishing it from the other population varieties with the exception of the varieties Chemlal Eloued, Chemlal Setif, and Sigoise Setif which showed very close characters.

## 4 CONCLUSION

The physicochemical parameters among geographical areas have revealed that both oils cultivars were enriched in pigments, tocopherols, and essential fatty acids. We found that all analyzed oils had a fatty acid composition in accordance with the marketing standard except a higher value of the linolenic acid recorded for the Sigoise cultivar oil from the Eloued area (1.34 ± 0.03) > 1.

The undertaken comparative description combined to a statistical data analysis revealed that variability depends on the measured parameters. Except for fatty acid composition, the geographical location was the prevailing factor, acting solely or in interaction with cultivar factor. Oil parameters that were affected only by the geographical location (yield, free fatty acids, total polyphenols, total tocopherols, and the specific extinction coefficient K270) seemed to be commonly regulated by the pedologic and climatic conditions (Table 1) of the cultivation area. Oil pigment contents, PV, and the RI were the most sensitive parameters, displaying fast changes in response to both geographical location and cultivar and their interactions. Fatty acid profiles were exclusively affected by cultivar, suggesting a genetic control on fatty acid composition of the analyzed olive oils.

## CONFLICT OF INTEREST

The authors confirm that they have no conflicts of interest with respect to the work described in this manuscript.

## STUDIES INVOLVING HUMAN OR ANIMAL SUBJECTS

Authors confirm that the present study experiments did not involve any human or animal subjects.

## Data Availability

The data that support the findings of this study are available on request from the corresponding author.
